# CD163+ macrophages are associated with a vulnerable plaque phenotype in human carotid plaques

**DOI:** 10.1038/s41598-020-71110-x

**Published:** 2020-09-01

**Authors:** Eva Bengtsson, Karin Hultman, Andreas Edsfeldt, Ana Persson, Mihaela Nitulescu, Jan Nilsson, Isabel Gonçalves, Harry Björkbacka

**Affiliations:** 1 Department of Clinical Sciences Malmö, Lund University, Malmö, Sweden; 2 Present Address: Innovation Department, Novo Nordisk Foundation, Hellerup, Denmark; 3 Department of Cardiology, Skåne University Hospital, Malmö, Sweden; 4 Wallenberg Center for Molecular Medicine, Lund University, Lund, Sweden

**Keywords:** Medical research, Molecular medicine

## Abstract

Macrophages are a functionally heterogeneous group of immune cells abundant in atherosclerotic plaques. Macrophages expressing CD163 are associated with intraplaque hemorrhage and have previously been considered atheroprotective. However, in a recent study CD163-deficient atherosclerotic *ApoE*^−/−^ mice exhibited smaller and less complex plaques, suggesting a proatherogenic role of CD163. Previous smaller studies on CD163+ macrophages and plaque stability in humans have yielded diverging results. Here we assessed the association of CD163+ cells to plaque vulnerability in a large cohort of human carotid plaques. CD163 protein expression was analyzed by immunohistochemistry in 200 human carotid plaques removed by endarterectomy from 103 patients with and 93 patients without cerebrovascular symptoms. Furthermore, CD163 mRNA expression was analyzed in 66 of the plaques. Both protein and mRNA expression of CD163 was higher in plaques from symptomatic patients and in plaques with high vulnerability index. CD163+ macrophages were primarily found in shoulder regions and in the center of the plaques. The present data show that CD163 is associated with increased plaque vulnerability in human carotid plaques, supporting the notion that CD163+ macrophages could contribute to clinical events.

## Introduction

Rupture of atherosclerotic plaques are responsible for the majority of ischemic strokes and myocardial infarctions^1,2^. Vulnerable or rupture-prone plaques are characterized by a large necrotic core, inflammation and signs of impaired tissue repair^3^. Plaque macrophages are a phenotypically heterogeneous group, where polarized phenotypes range from classically activated inflammatory (M1) macrophages to alternatively activated reparatory (M2) macrophages. Inflammatory macrophages are considered proatherogenic by their ability to take up lipids and become foam cells, as well as their ability to secrete inflammatory cytokines. In contrast, the alternatively activated macrophages are considered antiatherogenic by producing anti-inflammatory cytokines and promoting tissue repair^4^. Modulation of macrophages to a less inflammatory but a more reparative phenotype is a possible target in future plaque stabilizing treatments^5^.

The subtype of alternatively activated macrophages called Mhem/M(Hb) macrophages is characterized by the expression of CD163, a scavenger receptor for hemoglobin-haptoglobin complexes exclusively expressed on monocytes and macrophages^4^. CD163+ macrophages were first described in areas with intraplaque hemorrhage^6^, the latter contributing to plaque progression and destabilization^7^. In spite of this, CD163+ macrophages were considered atheroprotective due to their high expression of the anti-inflammatory cytokine IL-10 and heme degrading enzyme hemeoxygenase, as well as their association with reduced oxidative stress^6,8^. Furthermore, hemoglobin-haptoglobin or heme stimulated macrophages upregulate genes involved in reversed cholesterol transport, which further corroborated their role as atheroprotective macrophages^9,10^. Surprisingly, it was recently shown that CD163-deficient atherosclerotic *Apoe*^−/−^ mice exhibited increased plaque areas, less complex lesions, and thicker fibrous caps^11^. In addition, CD163+ macrophages were associated with plaque progression and angiogenesis in distal and proximal regions of 38 human carotid plaques^11^.

So far studies of CD163+ macrophages and plaque stability in human specimens have yielded diverging results^12–18^. However, these studies were relatively small. To address the question if CD163+ macrophages are associated with a stable or vulnerable plaque phenotype, we assessed CD163 expression by immunohistochemistry in a large cohort consisting of 200 carotid plaques removed by endarterectomy. CD163 expression was analyzed in relation to cerebrovascular symptoms, plaque vulnerability, and plaque components.

## Results

### CD163 is present in shoulder regions of human carotid plaques

**Figure 1:**
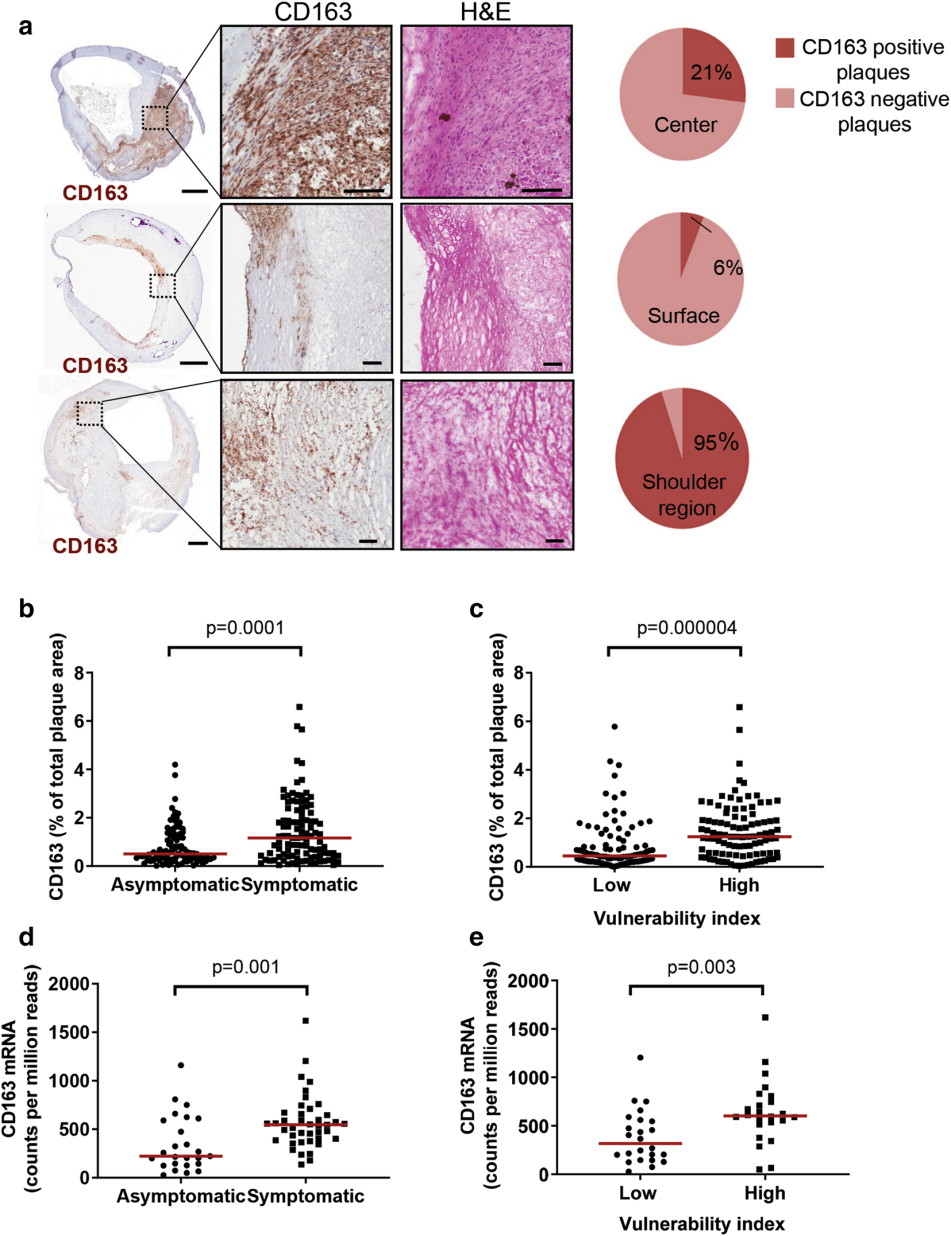
CD163 is present in shoulder regions of human carotid plaques, and CD163 expression is higher in plaques from symptomatic patients and in vulnerable plaques. Plaques (n = 200) from the carotid artery were stained with an anti-CD163 antibody and CD163 localization (% of plaques stained) in different plaque regions (**a**), and CD163 staining (% of total plaque area) were analyzed (**b**, **c**). Adjacent sections of hematoxylin eosin (H&E) staining of the individual plaques are shown in **a**. Pie charts indicate percentages of plaques with a positive CD163 staining in center, surface, or shoulder regions of the plaques (**a**). CD163 mRNA expression were determined in 66 human carotid plaques (**d**, **e**). Values are presented as individual values, and medians are indicated in red (**b**, **c**). The vulnerability index was determined by the sum of lipids (Oil Red O), macrophages (CD68), and hemorrhage (glycophorin A) divided by the sum of collagen (Movat pentachrome) and SMCs (α-actin) (**c**). Scale bars are 1 mm (plaques) or 100 µm (magnified parts of plaques).

The clinical characteristics of the patients are shown in Table 1. Expression of CD163 protein was most frequently observed in plaque shoulder regions and in the center of the plaque (Fig. 1a), whereas most surface regions were devoid of CD163 expression (Fig. 1a). A comparison between immunostainings of adjacent tissue sections revealed that CD163 staining was present in lipid- and macrophage-rich regions (Supplementary Figure 1). Furthermore, CD163 protein expression was increased in plaques with high intraplaque hemorrhage, but decreased in plaques enriched in calcification. There was also a trend to increased CD163 protein expression in lipid-rich plaques, whereas no differences were seen in CD163 protein expression in plaques with a high content of collagen or CD68, a general macrophage marker present on both M1 and M2 macrophages, or collagen (Supplementary Figure 2). CD163 staining was found in the same region as CD206 staining, a marker of M2 macrophages (Supplementary Figure 3A). In addition, CD163 was also to some extent found in the same region as the M1 macrophage and dendritic cell marker CD11c (Supplementary Figure 3B), similarly to what has been previously found in carotid endarterectomies by Cho et al.^14^.

**Table 1:** Clinical characteristics of the patient cohort.

	Total (n = 196)	Asymptomatic (n = 93)	Symptomatic (n = 103)	*p*
Age, years	69.3 (SD 8.4)	67.3 (SD 6.5)	71.2 (SD 9.5)	0.001
BMI, kg/m^2^	26.7 (SD 4.1)	26.9 (SD 4.0)	26.6 (SD 4.2)	0.62
Gender, % males	68 (n = 133 males)	68 (n = 63 males)	68 (n = 70 males)	0.97
Degree of stenosis, %	90 (IQR 80–95)	90 (IQR 80–95)	85 (IQR 75–95)	0.021
Diabetes, %	33 (n = 65)	25 (n = 23)	41 (n = 42)	0.017
Smoking (current), %	34 (n = 66)	39 (n = 36)	29 (n = 30)	0.16
Dyslipidemia, %	95 (n = 186)	98 (n = 91)	92 (n = 95)	0.12
**Fasting lipoproteins, mmol/L**				
Total cholesterol	4.4 (SD 1.1)	4.3 (SD 1.1)	4.4 (SD 1.2)	0.62
LDL cholesterol	2.4 (IQR 1.9–3.1)	2.2 (IQR 1.8–3.1)	2.6 (IQR 2.0–3.3)	0.15
HDL cholesterol	1.1 (IQR 0.9–1.3)	1.1 (IQR 0.9–1.4)	1.1 (IQR 0.9–1.2)	0.72
Triglycerides	1.3 (IQR 0.9–1.8)	1.3 (IQR 0.9–1.8)	1.2 (IQR 1.0–1.7)	0.54
Hemoglobin, g/L	141 (SD 13)	142 (SD 13)	140 (SD 13)	0.15
eGFR	78 (SD 28)	82 (SD 26)	74 (SD 29)	0.055
CRP, mg/L	3.9 (IQR 2.0–6.3)	3.7 (IQR 1.8–5.7)	4.0 (IQR 2.0–6.9)	0.26
White blood cell count, 10^9^/L	7.9 (SD 1.9)	7.9 (SD 1.9)	8.0 (SD 2.0)	0.72
Statins, %	87 (n = 171)	91 (n = 85)	84 (n = 86)	0.098
Anti-hypertensive treatment, %	81 (n = 158)	84 (n = 78)	78 (n = 80)	0.27

Values are presented as mean and standard deviation (SD), or when not normally distributed as median with interquartile range (IQR). *p* represents the significance comparing the symptomatic and asymptomatic patient groups. *BMI* body mass index, *CRP* high sensitivity C-reactive protein, *eGFR* estimated glomerular filtration rate, *HDL* high-density lipoprotein, *IQR* interquartile range, *LDL* low-density lipoprotein.

### CD163 is associated with a vulnerable plaque phenotype

To investigate the association between CD163 and plaque stability we compared the CD163 expression in lesions from patients with (n = 103) and without (n = 93) cerebrovascular symptoms. The expression of CD163 protein was significantly higher in lesions from symptomatic patients compared with asymptomatic patients (Fig. 1b). Furthermore, CD163 protein expression was higher in lesions with a high vulnerability index (Fig. 1c). CD163 mRNA expression were higher in plaques from symptomatic patients as well as in plaques with a high vulnerability index (Fig. 1d,e). Plaques from symptomatic patients were more vulnerable than plaques from asymptomatic patients (Supplementary Figure 4). To test if there were any relationships, not necessarily linear, between CD163 and individual plaque components Spearman rank correlation analysis was performed. Weak increasing trends (positive Spearman rank correlation coefficients) were found between CD163 protein and macrophages, lipids, glycophorin A, matrix metalloproteinase (MMP)-1, -9, -10 and TIMP-1, whereas weak decreasing trends (negative Spearman rank correlation coefficients) were found between CD163 protein and SMCs, elastin, collagen, and MMP-2 (Table 2). CD163 protein expression did not associate with time between symptoms and removal of the plaque (r =  − 0.031, *p* = 0.75) (mean time between symptoms and operation was 14 days). CD163 mRNA expression displayed a weak increasing trend to macrophages, and moderate increasing trends to lipids and intraplaque hemorrhage, as well as to MMP-1 and MMP-9 (Table 2).

**Table 2:** Spearman rank correlation coefficients between CD163 and plaque structural components, MMPs, and TIMPs in human carotid plaques.

	CD163 (% of plaque area)^1^ r-value	CD163 (mRNA)^4^ r-value
**Plaque structural components**		
Macrophages (CD68)^1^	0.182*	0.27*
Smooth muscle cells (α-actin)^1^	− 0.268***	− 0.24
Elastin^2^	− 0.270***	− 0.084
Collagen^2^	− 0.232**	− 0.13
Lipids (ORO)^1^	0.229**	0.37**
Hemorrhage (Glycophorin A)^1^	0.260***	0.52***
**MMPs and TIMPs**		
MMP-1^3^	0.199**	0.45**
MMP-2^3^	− 0.157*	0.057
MMP-3^3^	− 0.136	0.10
MMP-9^3^	0.224**	0.61***
MMP-10^3^	0.160*	0.20
TIMP-1^3^	0.453***	0.22
TIMP-2^3^	0.059	− 0.12

^1^Percentage staining of plaque area, ^2^Milligram per g plaque wet weight, ^3^Picogram per g plaque wet weight, ^4^counts per million read.

Next, we asked if CD163 correlations to plaque components were dependent on the plaque composition, e.g. if CD163 correlations to plaque components differed in highly calcified plaques compared to correlations performed in the total plaque cohort. CD163 correlations to plaque components were analyzed specifically in plaques enriched in calcification, hemorrhage, lipids, macrophages, or collagen. However, CD163 correlations to most of the stabilizing and destabilizing plaque components were about the same regardless of if the plaques were characterized by a high content of some specific plaque component (Supplementary Figure 5).

### CD163 is associated with proinflammatory cyto- and chemokines, and with type II cytokines

CD163 have previously been considered atheroprotective, partly due to in vitro studies showing that CD163 macrophages upregulate anti-inflammatory cytokines^8,10,19^. We therefore asked whether CD163 expression is associated with an anti- or pro-inflammatory cytokine profile in human plaques. Indeed, CD163 protein content was increased in plaques with high levels of IL-10 or IL-1-receptor antagonist (IL1-RA), whereas CD163 tended to be decreased in plaques with high levels of the type-I cytokines IL-12(p70) and IFN-γ (Table 3). In addition, CD163 protein was increased in plaques with a high content of the chemoattractants MCP-1 and MIP-1β, as well as pro-inflammatory cytokine IL-6.

**Table 3:** CD163 expression in plaques with low versus high levels of cytokines and chemoattractants.

		CD163^1^ Median (IQR)	*p* value
**Pro-inflammatory cytokines**			
IL-1β^2^	Low	0.55 (0.28–1.64)	0.13
	High	0.84(0.39–1.81)	
IL-6^2^	Low	0.55 (0.25–1.29)	0.010
	High	0.95 (0.36–1.89)	
TNF-α^2^	Low	0.70 (0.34–1.59)	0.82
	High	0.54 (0.23–1.43)	
**Type I cytokines**			
IL-12(p70)^2^	Low	1.10 (0.39–1.78)	0.045
	High	0.54 (0.23–1.43)	
IFN-γ^2^	Low	0.86 (0.40–1.83)	0.071
	High	0.55 (0.23–1.56)	
**Type II cytokines**			
IL-4^2^	Low	0.58 (0.29–1.54)	0.003
	High	1.34 (0.52–2.27)	
IL-10^2^	Low	0.53 (0.25–1.38)	0.003
	High	1.12 (0.48–1.92)	
IL-1RA^2^	Low	0.49 (0.22–1.07)	0.000005
	High	1.25 (0.45–2.17)	
**Monocyte chemoattractants**			
MCP-1^2^	Low	0.55 (0.23–1.54)	0.016
	High	0.87 (0.40–1.83)	
MIP-1β^2^	Low	0.54 (0.25–1.32)	0.021
	High	0.89 (0.39–1.85)	

*IL* interleukin, *IFN-gamma* interferon-gamma, *TNF-α* tumor necrosis factor-alpha.

IL-6, TNF-a, IL-12(p70), MCP-1, and MIP-1β were divided in below median (low) and above median (high). IL-1β, IL-4, and IL-10 were divided in detectable (high) and non-detectable (low). ^1^Percentage of plaque area, ^2^Picogram per g plaque wet weight.

### CD163 and association with cardiovascular risk factors

Plaque CD163 protein levels did not differ between smokers and non-smokers, but CD163 tended to be higher in men compared to women (Supplementary Table 1), the latter is in agreement with previous data from Yuan et al.^20^. There were no significant differences in CD163 protein expression between lesions from diabetic, hypertensive, or statin-treated patients compared to non-affected/non-treated patients. CD163 did not associate with body mass index, cholesterol, triglycerides, LDL, CRP, white blood cell count, or estimated glomerular filtration rate (eGFR) in blood. However, plaque CD163 content tended to be inversely correlated with HDL cholesterol (Supplementary Table 2).

## Discussion

In the present study we show that (i) CD163+ macrophages are present in shoulder regions of human carotid plaques, (ii) the levels of CD163+ macrophages and CD163 mRNA are higher in plaques from patients with cerebrovascular symptoms and in vulnerable plaques, and (iii) CD163+ macrophages are positively correlated with markers associated with plaque vulnerability, but negatively correlated with markers associated with plaque stability. Taken together, these results indicate that CD163 is associated with a vulnerable plaque phenotype in humans.

Macrophages are highly plastic cells that can adapt to perform many functions in response to environmental cues. At opposite ends of this functional spectra researchers have placed the inflammatory (M1) and repair- and resolution-associated macrophages (M2). The placement of CD163-expressing macrophages along this, or some other orthogonal, axis is still debated. Although single-cell RNA sequencing studies of human plaques are still in their infancy, they could shed light on the macrophage subpopulations present in atherosclerotic plaques. Fernandez et al. recently reported on a meta cluster of macrophages with enriched expression of both CD206 and CD163, as well as a meta cluster of macrophages with less, but not absent, expression of both CD206 and CD163 revealed by using mass cytometry and single-cell RNA sequencing in human plaques^21^. Notably, both macrophage meta clusters seemed to express CD11c. CD11c has also been considered to be an M1 macrophage marker^14^. In mice, Cochain et al. identified five monocyte/macrophage populations by single-cell RNA sequencing, including the “resident-like macrophages” that contained macrophages expressing CD163 and CD206^22^. In conclusion, there are still several issues that need to be addressed before macrophage subpopulations in human plaques have been clearly defined, such as optimizing release of cells by digestive enzymes while preserving cell surface markers to allow enough cells to be analyzed from many plaques at adequate sequencing depths.

Previous studies of human atherosclerotic plaques have reported divergent results regarding CD163 expression and symptoms associated with plaque rupture. In some of these studies, increased CD163 expression was shown in carotid plaques from symptomatic patients^12,13^ and in unstable plaque segments^16,17^, whereas Medbury et al. found no association between CD163 levels and plaque stability^15^. However, the number of plaques or plaque segments analyzed did not exceed more than 40 in any of the studies. In a slightly larger study consisting of 67 coronary plaques from patients with unstable or stable angina, a higher CD163 expression was observed in patients with unstable angina^18^. In contrast, Cho et al. reported increased CD163 expression in carotid plaques from 34 asymptomatic patients compared to 31 symptomatic patients^14^. The reasons for the discrepancy between these previous findings can only be speculated about, but several components may influence the results of the studies. For example, the location of the plaque (carotid artery or coronary artery) or the method of CD163 analysis (mRNA or protein expression) differ between the studies. Furthermore, the time between symptoms and endarterectomy may vary between the studies. The latter is of importance, since healing processes induced after plaque rupture could affect the result. Finally, the size of the cohort influences the statistical power where smaller studies may be underpowered to reveal differences. In the present paper, we analyzed CD163 expression in a considerably larger cohort compared to previous studies. Furthermore, the mean time between symptoms and removal of the plaque in the present cohort was short, and CD163 expression did not correlate with the number of days between symptoms and removal of the plaque, arguing that the present finding of increased CD163 expression in symptomatic plaques are not due to repair responses induced after the plaque rupture.

Consistent with CD163 being a scavenger receptor for hemoglobin-haptoglobin, CD163+ macrophages have previously been shown to present at sites of intraplaque hemorrhage^6,10^ and to correlate with glycophorin A^18^, present on erythrocytes. In accordance, we found an association between the plaque levels of CD163 protein or mRNA and glycophorin A in the present study. Interestingly, we also found a positive association of CD163+ cells with lipids, but a negative association with collagen, elastin, and SMCs. These data are in line with a previous study by Kolodgie et al. who reported that injection of erythrocytes into rabbit atheromas increased macrophage infiltration and lipid accumulation (determined by oil red O staining)^7^. Furthermore, the same authors found markedly increased glycophorin A levels in cholesterol clefts, as well as correlations between glycophorin A, macrophage content and necrotic core size in human coronary lesions. The authors concluded that intraplaque hemorrhage and the resulting accumulation of erythrocyte membranes, rich in cholesterol, contribute to cholesterol accumulation, macrophage infiltration, and an increased necrotic core in atherosclerotic plaques^7^. It is conceivable that the correlation of CD163 and lipids observed in the present study reflects plaque accumulation of cholesterol and hemoglobin initially derived from erythrocytes, and a subsequent accumulation of lipids and polarization of infiltrating monocytes towards the Hem/Mhem phenotype. Although the mechanisms behind hemorrhage induced infiltration of inflammatory cells was not elucidated by Kolodgie et al., it has been suggested that monocyte infiltration could be stimulated by chemoattractants bound to receptors on erythrocyte membranes, including MCP-1^23^. Interestingly, we found an association between CD163 levels and the monocyte chemoattractants MCP-1 and MIP-1β.

Excess free cholesterol in macrophages can disrupt membranes and is cytotoxic, and is therefore either transported out of the cell via cholesterol efflux and reverse cholesterol transport to the liver, or stored as cholesterol esters in lipid droplets^24^. Although, uptake of oxidized LDL in macrophages resulting in foam cell formation have been considered inflammatory, other papers show that macrophage foam cell formation in vivo in mice exhibit reduced expression proinflammatory mediators^25^ and more profibrotic phenotype^26^. However, in the current paper we cannot say if the correlation between CD163 and lipids reflects extracellular or intracellular lipids.

Previous studies link CD163 to anti-inflammatory cytokines: CD163 expression in monocytes is upregulated upon stimulation with IL-10, whereas treatment with IFN-γ and TNF-α downregulates CD163^27^. Furthermore, CD163^high^ macrophages are potent IL-10, and IL-1RA producers^8,10^, and have been associated with IL-10 content of coronary atherectomy specimens from patients with stable or unstable angina^18^. In agreement, the present paper show statistically significant correlations between high CD163 and high levels of anti-inflammatory IL-10 and IL-1RA in the plaques, whereas CD163 levels were lower in plaques with a high expression of type I cytokines (IL-12p70 and IFNγ). However, we also found that CD163 correlated positively with plaque content of proinflammatory IL-6, and the chemokines MCP-1 and MIP-1β. This is consistent with previous studies, which in addition to increased IL-10, show increased MCP-1 secretion by CD163^high^ macrophages^10^. Furthermore, CD163 expression was upregulated in IL-6 stimulated monocytes^27^.

The present study includes a number of limitations, which need to be acknowledged. Importantly, since this an associative study, conclusions regarding the causality of CD163 and plaque vulnerability or plaque components in human plaques cannot be drawn. Second, CD163 protein expression was analyzed in the most stenotic part of the plaque, and it is possible that the CD163 expression is different in other parts of the plaque. Furthermore, all cases may not have a typical necrotic core in the most stenotic region of the plaques. These limitations are important to take into consideration in the comparisons between CD163 protein expression and plaque localization, or between CD163 protein expression determined in sections from the most stenotic part and cytokine levels determined in plaque homogenates from other parts of the plaque. Third, only advanced plaques with > 70% stenosis were analyzed, and CD163 expression in these plaques may differ compared to smaller plaques. Fourth, the correlations between CD163 expression and plaque components are in most cases, statistically speaking, weak. The current study, however, analyses CD163 in a large cohort of 200 human carotid plaques, removed from both symptomatic and asymptomatic patients. These plaques were extensively characterized for plaque structural and inflammatory components. Together this enables a thorough analysis of CD163 to plaque vulnerability.

In conclusion, we show that CD163+ macrophages are associated with a vulnerable plaque phenotype in human carotid plaques, which supports the notion that CD163 could contribute to clinical events.

## Methods

### Patients and sample preparation

Carotid plaques (n = 200) were collected from 196 individuals at carotid endarterectomy. Four patients underwent carotid endarterectomy at two separate time points The indicators for surgery were (i) plaques associated with ipsilateral symptoms (transient ischemic attack, stroke, or amaurosis fugax) and with > 70% stenosis measured by duplex, (n = 97), or (ii) plaques not associated with symptoms, but with > 80% stenosis (n = 103). Written and oral informed consent was given by each patient one day before surgery. This cross-sectional study was approved by the Regional Ethics Committee in Lund (Approval No. 472/2005). The clinical characteristics of the patients are summarized in Table 1. All patients were preoperatively assessed by a neurologist. After surgical removal, the carotid plaques were snap-frozen in liquid nitrogen and 1-mm thick fragments were cut from the most stenotic region of the plaque. Plaque homogenates were prepared as previously described^28^. One day before surgery a blood sample was taken and analyzed for fasting lipoproteins, hemoglobin, creatinine, CRP, and white blood cell content. All methods were carried out in accordance with relevant guidelines and regulations.

### Immunohistochemistry

Transversal tissue section (8 µm thick) from the plaque fragments were stained with immunohistochemistry for vascular SMCs (α-actin), macrophages (CD68), CD163, glycophorin A, and lipids. For staining of α-actin, CD68, CD163, CD11c, and glycophorin sections were fixed in acetone, permeabilized with 0.5% Triton-X, and incubated in phosphate-buffered saline (PBS) or methanol containing 3% H_2_O_2_ to neutralize endogenous peroxidase activity. After pre-incubation in PBS containing 10% serum or 10% bovine serum albumin, sections were incubated with either CD163 antibody (ab15676, or ab156769 Abcam), α-actin antibody (clone 1A4, DakoCytomation, Glostrup, Denmark), CD68 antibody (clone PG-M1, DakoCytomation), glycophorin A antibody (CD235a, clone JC159, DakoCytomation), CD11c antibody (ab11029, Abcam) or IgG isotype control antibodies (sc-2025, Santa Cruz Biotechnology, or ab27478, ab81032, Abcam) over night. After washing, sections were incubated with secondary biotinylated antibodies (goat anti-rabbit, Vector Laboratories or rabbit anti-mouse, DakoCytomation) diluted in PBS and 10% serum for 1 h followed by Vectastain ABC kit (Vector Laboratories), or with MACH3 mouse probe (Biocare Medical) followed by MACH3 mouse HRP-polymer (Biocare Medical). Sections were washed and developed using DAB peroxidase substrate kit (ImmPACT DAB, Vector Laboratories). After color development slides were immediately washed under tap water. Counterstaining was achieved using Mayer’s hematoxylin. Slides were mounted with VectaMount (Vector Laboratories) and observed under a light microscope. Specificity of immune staining was confirmed by the absence of staining in sections incubated with IgG isotype control antibodies or exclusion of primary antibodies. CD206 (ab64493, Abcam) staining was performed as above, but without permeabilisation with Triton X-100. Lipids were determined by Oil Red O staining as previously described^29^. Calcium was determined by von Kossa staining. Sections were hematoxylin–eosin stained with Mayers hematoxylin for 4 min, rinsed in water, followed by 1% erythrosine, and finally rinsed in water. Areas of the different stainings in the plaques (% of total plaque area) were quantified blindly using Biopix iQ 2.1.8 (Gothenburg, Sweden) after scanning with Scansope Console Version 8.2 (LRI imaging AB, Vista California, USA) and photographed with Aperio image scope v.8.0 (Aperio, Visat California, USA). The vulnerability index of each plaque was determined by the sum of lipids (Oil Red O), macrophage (CD68), and hemorrhage (glycophorin A) divided by the sum of collagen (Movat pentachrome) and SMCs (α-actin) as described^30,31^, all determined as percentage staining of total plaque area. Vulnerability index was divided in low (below median) and high (above median).

### Assessment of collagen, elastin, cytokines, and MMPs and TIMPs in plaque homogenates

Collagen was measured by Sircol soluble Collagen assay (Biocolor, UK) and elastin was measured by Fastin Elastin Assay (Biocolor). IL-1β, IL-6, TNF-α, IL-4, IL-10, IFN-γ, MCP-1, and MIP-1β were analyzed using human cytokine/chemokine immunoassay (Millipore Corporation, MA, USA) and analyzed with Luminex 100 IS 2.3 (Austin, Tx, USA). MMP-1, -2, -3, -9, -10 were analyzed using Mesoscale human MMP ultra-sensitive kit (Mesoscale, Gaithersburg, MD, UDA). TIMP-1 and TIMP-2 were analyzed using Milliplex MAP Human TIMP Magnetic Bead Panel (Milliplex, MA, USA). All analyses were normalized against wet weight of the plaque.

### Assessment of CD163 mRNA in plaque homogenates

The gene expression of CD163 was assessed in plaque tissue of 66 carotid plaques from a global transcriptome RNA sequencing data. Libraries were sequenced on a Illumina HiSeq2000 platform as previously described^32^.

### Statistics

Variables are presented as mean (standard deviation, SD), median (interquartile range), or percentages. Two-groups comparisons of normally distributed data were analyzed by *t*-test and non-normally distributed variables were analyzed by Mann–Whitney test. Cytokine levels were divided in below and above median, or above and below detection limit. Spearman rho was used to determine correlations. Significance was considered at *p* < 0.05. IBM SPSS statistics 24 was used for the statistical analysis.

## Supplementary information

Supplementary file1

## Data Availability

The dataset generated during and/or analysed during the current study are not publicly available due to ethical legislation, but will be available from the corresponding author on reasonable requests complying to ethical legislation.
