# Supplementary material for: CD163+ macrophages are associated with a vulnerable plaque phenotype in human carotid plaques
Source: Sci Rep. 2020 Sep 1;10:14362. doi: 10.1038/s41598-020-71110-x (PMC7463157; doi:10.1038/s41598-020-71110-x)
Supplement: Supplementary file 1 — Supplementary file1 [file 41598_2020_71110_MOESM1_ESM.pdf]

CD163+ macrophages are associated with a vulnerable plaque phenotype in human carotid plaques

Eva Bengtsson*[1], Karin Hultman*[2], Andreas Edsfeldt[1,3,4], Ana Persson[1], Mihaela Nitulescu[1], Jan Nilsson[1], Isabel Gonçalves†[1,3], Harry Björkbacka†[1]

***,† Contributed equally**

[1]Experimental Cardiovascular Research Unit, Department of Clinical Sciences, Malmö, Lund University, Sweden
[2]Present address: Novo Nordisk Foundation, Innovation Department, Hellerup, Denmark
[3]Department of Cardiology, Skåne University Hospital, Sweden
[4]Wallenberg Center for Molecular Medicine, Lund University, Sweden

Supplementary Table 1

Association of CD163 with risk factors or medication in patients.

		CD163[1] Median (IQR)	p-value
Gender	Male	0.84 (0.38-1.86)	0.080
	Female	0.59 (0.29-1.40)	
Smoking	No	0.72 (0.37-1.80)	0.68
	Yes	0.81 (0.29-1.65)	
Diabetes	No	0.75 (0.32-1.63)	0.59
	Yes	0.70 (0.38-1.79)	
Anti-hypertensive	No	1.18 (0.41-1.61)	0.30
	Yes	0.69 (0.32-1.77)	
Statins	No	0.71 (0.38-2.44)	0.31
	Yes	0.75 (0.32-1.63)	

[1]CD163 (% of plaque area) is presented as median (IQR).

Supplementary Table 2

Spearman rank correlations coefficients between CD163 expression in plaques and cardiovascular risk factors in patients.

	CD163	
	r	p-value
Age	0.099	0.17
Body mass index	-0.084	0.24
Fasting lipoproteins		
Total cholesterol	-0.059	0.43
LDL cholesterol	0.017	0.82
HDL cholesterol	-0.14	0.062
Triglycerides	0.0001	1.00
eGFR	-0.092	0.20
CRP	0.009	0.90
White blood cell count	0.026	0.72

eGFR (estimated glomerular filtration rate), CRP (high sensitivity C-reactive protein).

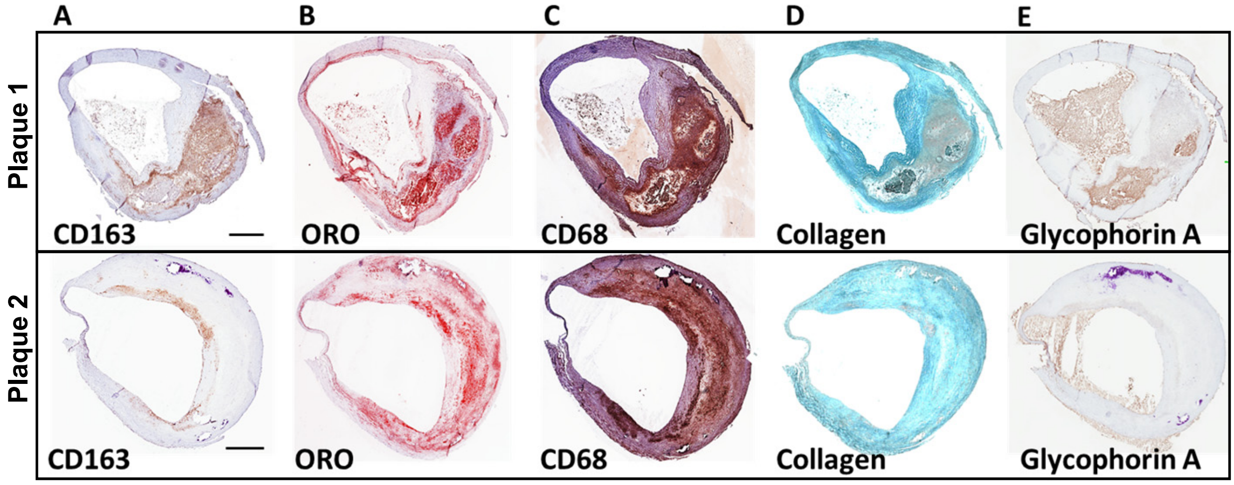

Supplementary figure 1.
Human plaques stained for CD163 (a), lipids (Oil red O) (b), CD68 (c), collagen (d), and glycophorin A (e). Representative images for two different plaques are shown. Scale bars = 1 mm.

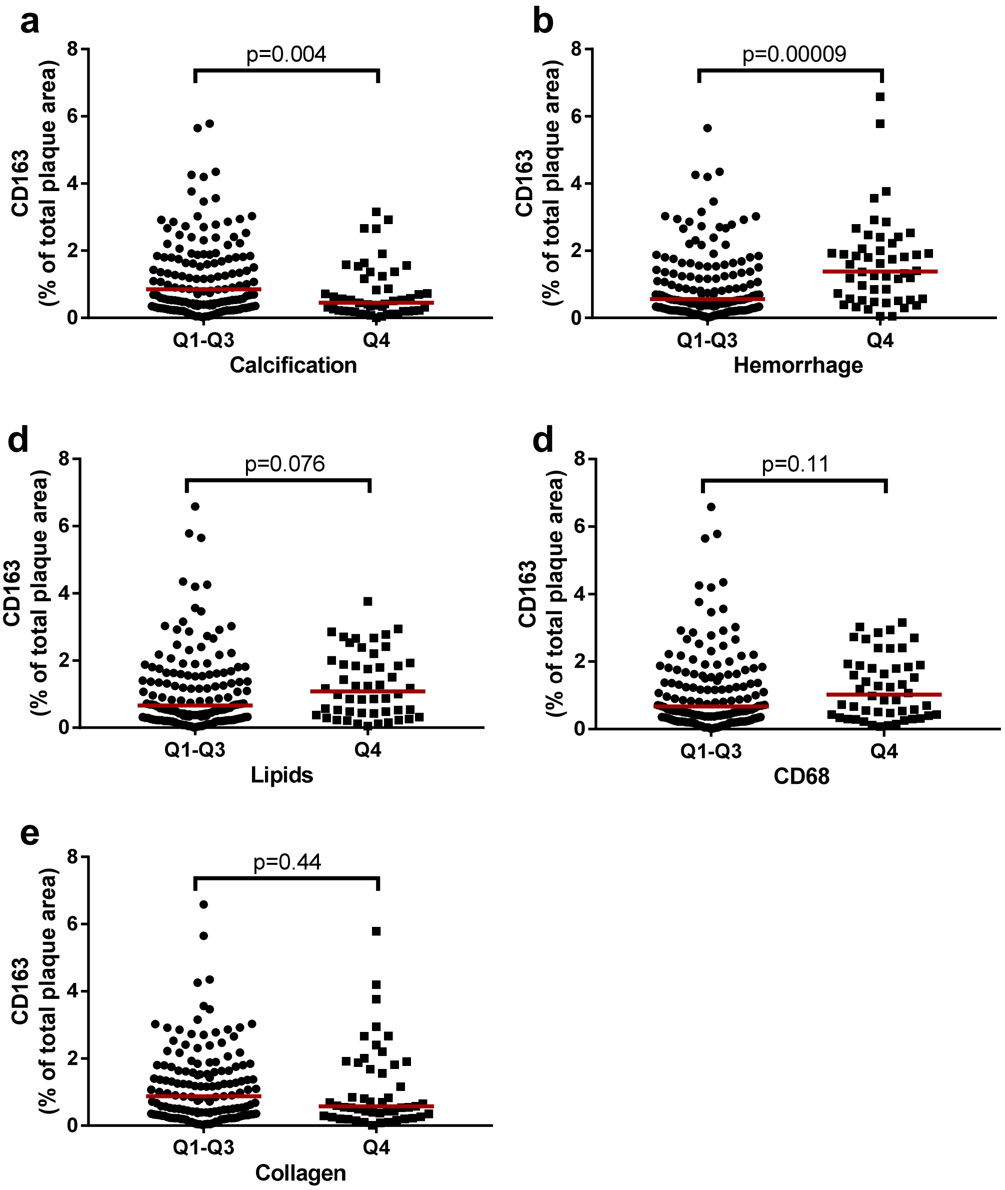

Supplementary figure 2.

CD163 protein expression in plaques enriched in calcification (a), hemorrhage (b), lipids (c), CD68 (d), or collagen (e).

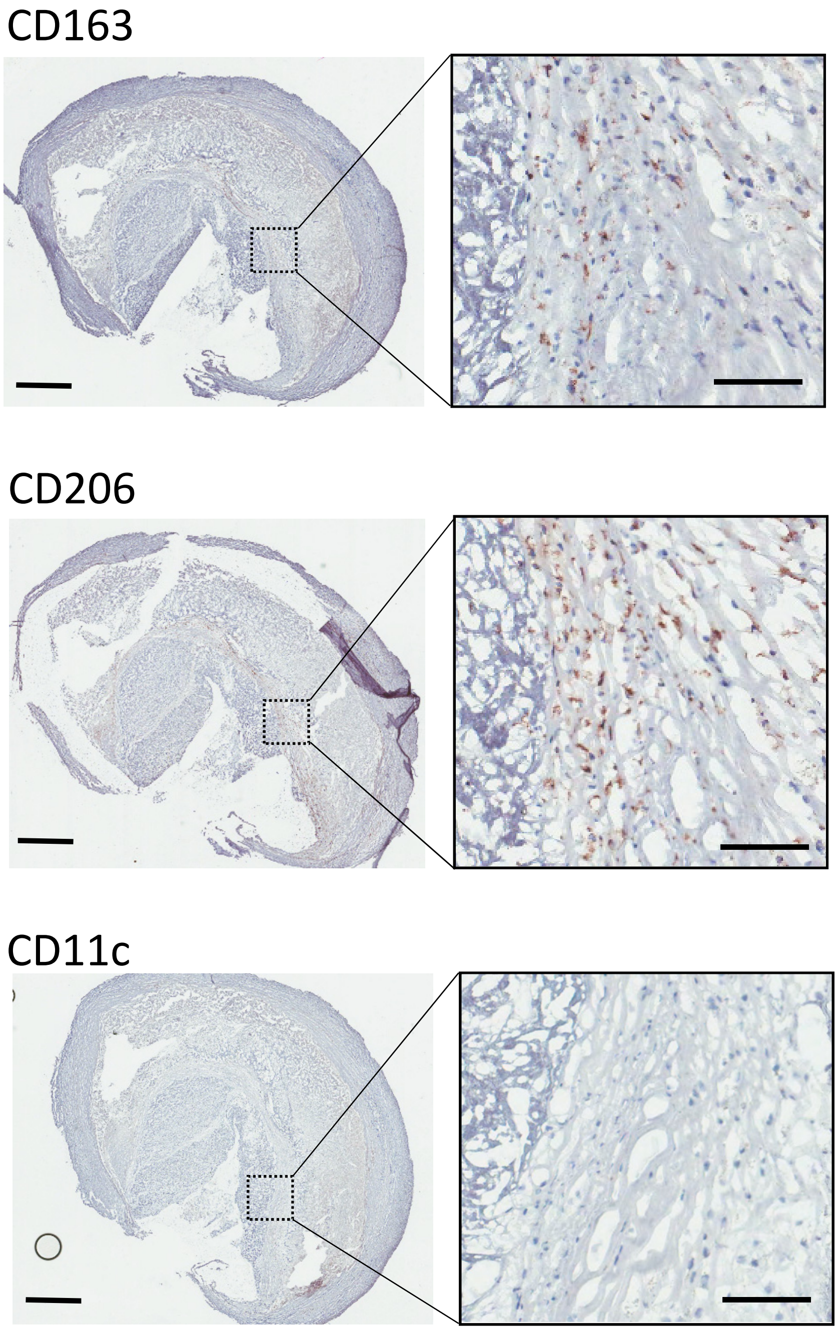

Supplementary figure 3a.

Human carotid plaque stained for CD163, CD206, and CD11c.

Scale bars are 1 mm (whole plaque) or 100 µm (magnified part of plaque).

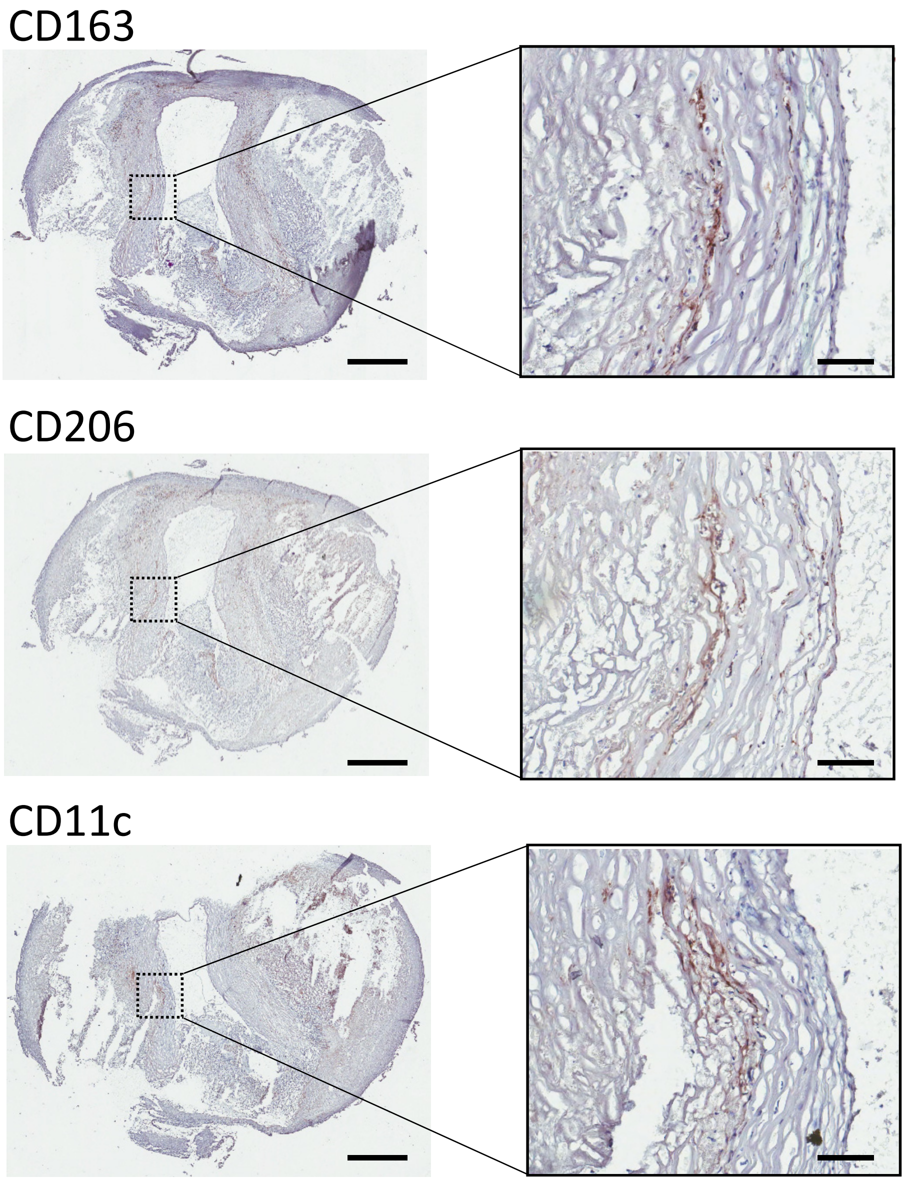

Supplementary figure 3b.
Human carotid plaque stained for CD163, CD206, and CD11c.
Scale bars are 1 mm (whole plaque) or 100 µm (magnified part of plaque).

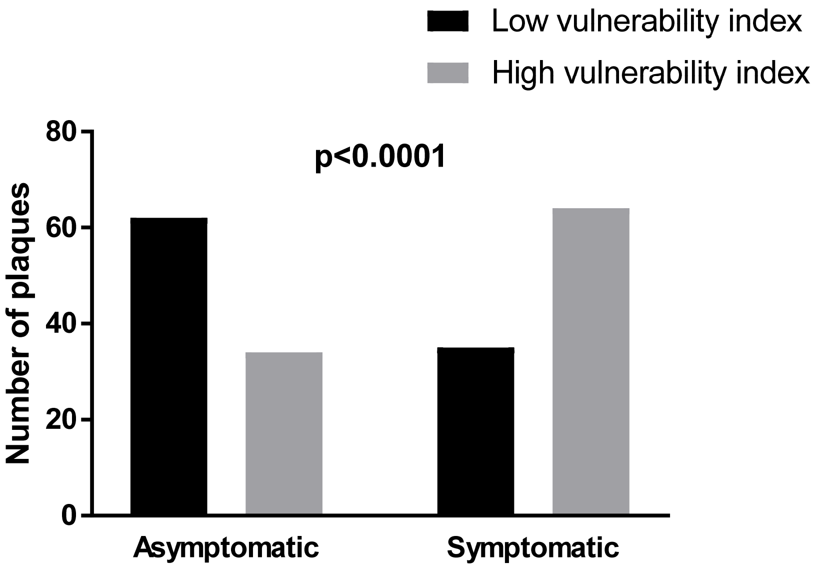

Supplementary figure 4.
Plaques from symptomatic patients have increased plaque vulnerability. The vulnerability index was determined by the sum of lipids (Oil Red O), macrophages (CD68), and hemorrhage (glycophorin A) divided by the sum of collagen (Movat pentachrome) and SMCs (α-actin).

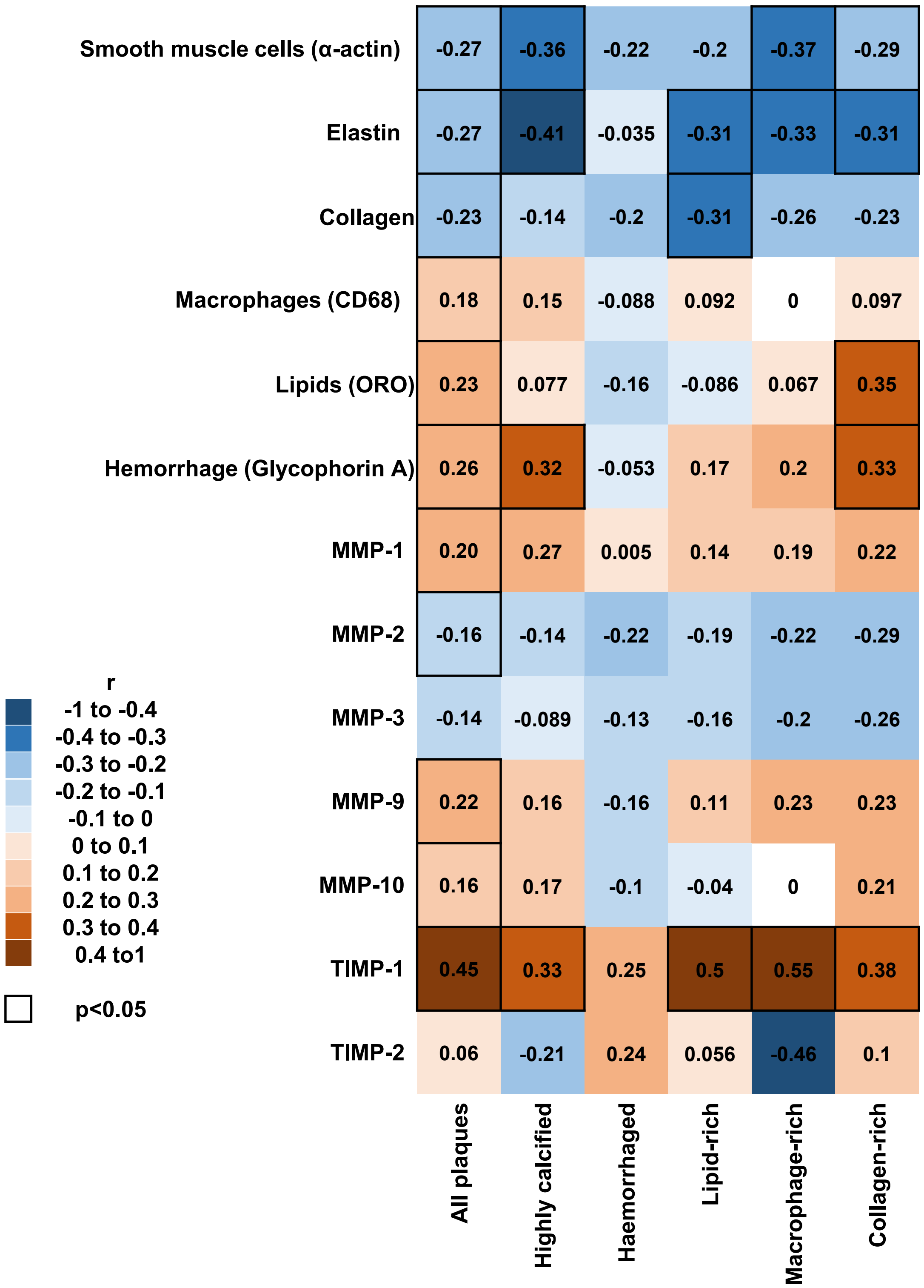

Supplementary figure 5.
Correlations between CD163 protein expression and plaque structural components, MMPs, and TIMPs in human carotid plaques rich in calcium, hemorrhage, lipids, macrophages and collagen. Spearman correlation coefficients (r) are shown in color coded squares. Statistically significant correlations (p<0.05) are marked with a border.
